# Additive Manufacturing: Alloy Design and Process Innovations

**DOI:** 10.3390/ma13030542

**Published:** 2020-01-23

**Authors:** Konda Gokuldoss Prashanth, Zhi Wang

**Affiliations:** 1 Department of Mechanical and Industrial Engineering, Tallinn University of Technology, Ehitajate Tee 5, 19086 Tallinn, Estonia; 2 Erich Schmid Institute of Materials Science, Austrian Academy of Science, Jahnstrasse 12, A-8700 Leoben, Austria; 3 CBCMT, School of Mechanical Engineering, Vellore Institute of Technology, Vellore 632014, Tamil Nadu, India; 4 School of Mechanical and Automotive Engineering, South China University of Technology, Guangzhou 510640, China; wangzhi@scut.edu.cn

Additive Manufacturing (AM) is an emerging manufacturing technique of immense engineering and scientific importance and is also regarded as the technique of the future. AM can fabricate any kind of materials including metals, polymers, ceramics, composites, etc. It also offers several advantages, like added functionality, offering intricacy for free, near-net-shape fabrication with minimal or no post-processing, shorter lead time, etc. AM has been used in several industrial sectors like aerospace, automobile, oil refinery, marine, construction, food industry, jewelry, etc. However, the several shortcomings in the field of AM are (a) alloy development that suits the AM processes (b) pre-mature failure of materials, even though improved properties are observed (c) process development and innovation (d) structure-property correlation, etc.

Accordingly, the present Special Issue (book) focuses on the two main aspects: alloy design and process development and innovation. Alloy design and development that suits the process conditions and the process is requisite, and the conventionally designed alloys for powder metallurgy/casting process are unusable. In addition, process development is happening at a rapid pace in the field of AM, which also warrants attention. Overall, 45 papers were published under this Special Issue with the following themes:

Selective Laser Melting/Laser-based powder bed fusion process of materials—23 papers including processing of Al-based alloys, Fe-based alloys, Ni-based alloys, Ti-based alloys and Zr-based alloys:

Direct Metal Laser Sintering—1 paper

Laser Cladding—3 papers

Electron Beam Melting—2 papers

Hybrid Manufacturing (Additive + Subtractive Manufacturing)—1 paper

Wire Arc Additive Manufacturing—3 papers

Fusion Deposition Modeling—2 papers

Ultrasonic Consolidation—1 paper

Miscellaneous fields—9 papers

The outcome of the Special Issue suggests that research is thriving in the field of AM, especially alloy design and process innovations. The present Special Issue is particularly interesting because it covers a wider range of AM processes and materials and gives an overview of research in this field, including alloy design and development, process development, microstructure-property correlation, simulation on melt pool dynamics, etc.

Finally, we would like to thank all the contributing authors for their excellent contribution to this Special Issue, to the reviewers for constructively improving the quality of the Special Issue and to the Materials staff for giving us the opportunity to host this Special Issue and to publish the articles in a timely manner and make this Special Issue a great success.

